# Comparison of platinum combination re-challenge therapy and docetaxel monotherapy in non-small cell lung cancer patients previously treated with platinum-based chemoradiotherapy

**DOI:** 10.1186/s40064-015-0929-3

**Published:** 2015-03-31

**Authors:** Hisao Imai, Kyoichi Kaira, Keita Mori, Akira Ono, Hiroaki Akamatsu, Tetsuhiko Taira, Reiko Yoshino, Hirotsugu Kenmotsu, Jun-ichi Saitoh, Hideyuki Harada, Tateaki Naito, Haruyasu Murakami, Yoshio Tomizawa, Masana Matsuura, Ryusei Saito, Takashi Nakajima, Masanobu Yamada, Toshiaki Takahashi

**Affiliations:** Division of Thoracic Oncology, Shizuoka Cancer Center, 1007 Shimonagakubo, Nagaizumi, Suntou-gun Shizuoka, 411-8777 Japan; Clinical Trial Coordination Office, Shizuoka Cancer Center, 1007 Shimonagakubo, Nagaizumi, Suntou-gun Shizuoka, 411-8777 Japan; Division of Radiation Oncology, Shizuoka Cancer Center, 1007 Shimonagakubo, Nagaizumi, Suntou-gun Shizuoka, 411-8777 Japan; Division of Diagnostic Pathology, Shizuoka Cancer Center, 1007 Shimonagakubo, Nagaizumi, Suntou-gun Shizuoka, 411-8777 Japan; Department of Respiratory Medicine, National Hospital Organization Nishigunma Hospital, 2854 Kanai, Shibukawa, Gunma 377-8511 Japan; Department of Radiology, National Hospital Organization Nishigunma Hospital, 2854 Kanai, Shibukawa, Gunma 377-8511 Japan; Department of Medicine and Molecular Science, Gunma University Graduate School of Medicine, 3-39-15, Showa-machi, Maebashi, Gunma 371-8511 Japan; Department of Oncology Clinical Development, Gunma University Graduate School of Medicine, 3-39-15, Showa-machi, Maebashi, Gunma 371-8511 Japan; Department of Radiation Oncology, Gunma University Graduate School of Medicine, 3-39-15, Showa-machi, Maebashi, Gunma 371-8511 Japan

**Keywords:** Platinum combination, Docetaxel, Non-small cell lung cancer, Chemoradiotherapy, Recurrence, Second-line chemotherapy

## Abstract

Platinum-based chemoradiotherapy (CRT) is a standard front-line treatment for locally advanced non-small cell lung cancer (NSCLC). However, no clinical trials have compared the efficacy and toxicity of platinum combination and docetaxel as subsequent re-challenge chemotherapies after cancer recurrence following CRT. This study aimed to evaluate the efficacy and toxicity of platinum combination chemotherapy versus docetaxel monotherapy in NSCLC patients previously treated with platinum-based CRT.

From September 2002 to December 2009, at three participating institutions, 24 patients with locally advanced NSCLC, who had previously received platinum-based CRT, were treated with platinum combination re-challenge therapy, whereas 61 received docetaxel monotherapy. We reviewed their medical charts to evaluate patient characteristics and data regarding treatment response, survival, and toxicity.

The response rates were 16.7% and 6.6% in the platinum combination chemotherapy and docetaxel monotherapy groups, respectively (*p* = 0.09), whereas disease control rates were 58.3% and 57.4%, respectively (*p* = 0.82). Progression-free survival was similar between the two groups (median, 4.2 vs. 2.3 months; hazard ratio [HR] = 0.81; 95% confidence interval [CI] = 0.51–1.29; *p* = 0.38), as was overall survival (median, 16.5 vs. 13.0 months; HR = 0.82; 95% CI = 0.47–1.41; *p* = 0.47). The incidence and severity of toxicity was also similar between the two groups. Hematological toxicity, particularly leukopenia and neutropenia, was more frequent in the docetaxel group.

Our results indicated that platinum combination re-challenge was equivalent to docetaxel for relapsed patients previously treated with platinum-based CRT.

## Introduction

In 2012, lung cancer was the most frequently diagnosed cancer and the leading cause of cancer-related death in men worldwide (Torre et al. 2015). Non-small cell lung cancer (NSCLC) accounts for 85% of all lung cancer cases, and more than 50% of NSCLC patients are diagnosed at advanced disease stages (Siegel et al. 2012). Stage III NSCLC is a heterogeneous disease, likely due to the involvement of different nodes, and as such, it is difficult to treat. Sause *et al.* reported that adding chemotherapy to radiotherapy prolonged survival (Sause et al. 2000). A recent meta-analysis concluded that concurrent chemoradiotherapy (CRT) was state-of-the art treatment for patients with NSCLC (Auperin et al. 2010), and CRT is currently recommended as the standard first-line treatment for locally advanced NSCLC. The median survival of patients with stage III NSCLC has recently been updated from 12 to 23.3 months in phase III trials (Hanna et al. 2008; Vokes et al. 2007). Although concurrent CRT provides a high rate of tumor response (60–70%), it does not necessarily lead to a cure. In fact, recent phase III trials of concurrent CRT have reported that two-thirds of patients who experience complete or partial response eventually relapse (Segawa et al. 2010; Yamamoto et al. 2010) and ultimately require systemic therapy.

The current curative treatment of previously untreated locoregional disease frequently involves the use of chemotherapy, usually platinum-based, either as an adjuvant after surgery or concomitantly with high-dose radiotherapy (Auperin et al. 2006). In theory, patients who are initially treated with a platinum agent as part of CRT and then relapse may have been left with a clonal population of platinum-resistant malignant cells (Huisman et al. 2000). Therefore, when subsequently re-treated with platinum-based chemotherapy, they may not achieve the same degree of benefit as those who receive platinum-based chemotherapy as their first-line treatment.

Several cytotoxic agents, such as docetaxel and pemetrexed, are useful as second- or third-line treatments for NSCLC. Docetaxel is a standard second-line chemotherapy regimen that is most widely used in Japan. A randomized phase III study comparing docetaxel and best supportive care demonstrated better overall survival (OS) for docetaxel patients (7.5 vs. 4.6 months, *p* = 0.047) (Shepherd et al. 2000). Since then, a number of other cytotoxic agents have been introduced as effective agents for second-line treatment (Fossella et al. 2000; Hanna et al. 2004; Ramlau et al. 2006). However, the impact of previous chemotherapy on the efficacy of subsequent chemotherapy has yet to be established. The effectiveness of re-administration of cytotoxic anti-cancer drugs has been reported in patients with small-cell lung cancer who respond to initial treatment with the same drugs (Ebi et al. 1997). Similarly, more recent studies have shown that in NSCLC patients who respond to an initial treatment with gefitinib, the disease could be successfully controlled by re-challenging with gefitinib (Kurata et al. 2004; Tomizawa et al. 2010).

However, the efficacy and safety of re-challenging with platinum-based therapy for relapsed patients who initially receive platinum-based CRT is unknown. Since many other salvage drugs are available, the administration of cisplatin- or carboplatin-based salvage therapy is uncommon, and potential cumulative toxicity with platinum re-challenging (e.g. neurotoxicity) is of concern. Furthermore, the use of platinum-based regimens as second-line treatment has not been shown to improve survival rates in randomized trials. To our knowledge, there is no prospective research comparing the survival benefit between platinum-based regimen re-challenge and docetaxel monotherapy. Additionally, no clinical trial has compared the efficacy and toxicity of platinum combination re-challenge therapy and docetaxel monotherapy as subsequent chemotherapy after cancer recurrence following CRT. In fact, evidence for patients with post-CRT cancer recurrence is lacking. This study aimed to evaluate the efficacy and toxicity of platinum combination chemotherapy in comparison with docetaxel monotherapy in NSCLC patients who had previously been treated with platinum-based CRT.

### Patients and methods

#### Patients

We retrospectively reviewed 85 consecutive patients with locally advanced NSCLC who had previously received platinum-based concurrent CRT at three institutions (Shizuoka Cancer Center, National Hospital Organization Nishi-gunma Hospital, and Gunma University Hospital) between September 2002 and December 2009. Of these, 24 were subsequently treated with platinum combination chemotherapy at relapse, whereas 61 received docetaxel monotherapy. Both time-to-event variables were censored by the study cut-off date (April 1, 2014). The eligibility criteria for this study were: (1) histologically or cytologically proven NSCLC; (2) age of ≤ 75 years at the time of front-line CRT; (3) Eastern Cooperative Oncology Group Performance Status (ECOG PS) of 0–2; (4) treated with curative thoracic radiotherapy of greater than 50 Gy concurrent with platinum-doublet chemotherapy; and (5) subsequently treated with platinum combination chemotherapy or docetaxel monotherapy after cancer recurrence. The following baseline pre-treatment demographic and prognostic information was obtained: sex, age, ECOG PS, clinical stage at the time of recurrence, histology, smoking history, number of treatment cycles, response to prior CRT, interval between the final administration of the previous chemotherapy and the start of subsequent chemotherapy, radiation dosage, and prior chemotherapy regimen. All patients underwent systematic evaluation and standardized staging procedures before the start of treatment. Clinical stage was assigned based on the results of physical examination, chest radiography, computed tomography (CT) scans of the chest and abdomen, CT or magnetic resonance imaging of the brain, and bone scintigraphy or positron emission tomography. Patients with distant metastases before receiving chemoradiotherapy were excluded from this analysis. Tumor stage was determined according to the classification system of the International Association for the Study of Lung Cancer. The histologic classification of a tumor was based on the World Health Organization criteria (Travis et al. 1999). This study was approved by the institutional review board of each participating institution. Written informed consent was not required owing to the study’s retrospective design.

#### Treatment methods

Radiotherapy was administered using 6- or 10-MV X-rays in 2-Gy fractions five times weekly. All patient treatment plans were designed using a three-dimensional treatment planning system. The gross tumor volume was delineated according to nodal involvement determined by CT. The clinical target volume was defined and contoured with 5–10 mm around the gross tumor volume and outlines of the regional lymph node areas, *i.e*., the ipsilateral hilum and the mediastinum. Planning target volume (PTV) 1 was comprised of the clinical target volume plus a 5–10 mm margin, whereas PTV2 included the gross tumor volume plus a 10-mm margin. An additional margin was added if necessary. Beam shaping was performed using a multileaf collimator. The standard of practice was to prescribe a treatment dose of 60 Gy to PTV2 and 40 Gy to PTV1. Other objectives were to restrict the relative volume of normal lung irradiation at a dose of >20 Gy (V20) to ≤35%, and to restrict the maximum spinal cord dose to <44 Gy. The dose was prescribed to the isocenter.

#### Subsequent chemotherapy after post-CRT recurrence

The decision to re-treat with a platinum-based chemotherapy and the choice of subsequent platinum-based chemotherapy regimen were determined by the treating physician. For patients re-treated with docetaxel monotherapy, docetaxel was administered at a dose of 60 mg/m^2^/week given every three weeks or longer. For both regimens, treatment changes such as dose reduction, dose skipping, or dose delay were decided by the treating physician.

#### Assessment of efficacy and toxicity and statistical analysis

Radiographic tumor responses were evaluated according to the Response Evaluation Criteria in Solid Tumors (Therasse et al. 2000). The Common Terminology Criteria for Adverse Events v3.0 was used to evaluate acute adverse events until four weeks after the final chemotherapy administration or a patient’s death. All statistical analyses were performed using JMP, version 9.0, for Windows (SAS Institute, Cary, NC, USA). OS was calculated from the start of the first chemotherapy cycle to the date of death from any cause or that of the last follow-up. OS was estimated using the Kaplan-Meier method. The Fisher’s exact test and the Wilcoxon rank-sum test were used to compare the mean values of the variables between the two groups. Statistical significance was set at *p* < 0.05.

## Results

### Patient characteristics

Patient characteristics are shown in Table 1. Of the enrolled patients, 24 received platinum combination chemotherapy as their subsequent chemotherapy, whereas 61 received docetaxel monotherapy. Among those treated with platinum combination chemotherapy, six received cisplatin, and 18 carboplatin. There were no statistically significant differences between the two groups in terms of sex, age, PS, clinical stage at the time of recurrence, histology, smoking history, number of treatment cycles, time since prior CRT, radiation dosage, or prior chemotherapy regimen. However, the re-challenge group tended to have more patients with adenocarcinoma than the docetaxel group, and there were statistically significant differences between the two groups in terms of response to prior CRT and number of regimens after progression following second-line chemotherapy. After progressing past second-line chemotherapy, 23 of the 85 patients did not receive further chemotherapy. The chemotherapy regimens provided after the second-line regimen are shown in Table 2. The median follow-up time at the study censoring date was 27.3 months (range, 7.4–105.9 months).

**Table 1:** **Baseline patient characteristics at the beginning of chemotherapy by treatment arm**

**Characteristics**	**Platinum combination therapy (n = 24)**	**Docetaxel monotherapy (n = 61)**	***p*** **value**
Sex			
Male	22	52	0.40^a^
Female	2	9	
Age, median (range), years	63.5 (46–74)	65 (42–77)	0.78^b^
Performance status			
0	9	23	0.24^a^
1	15	34	
2	0	4	
Clinical stage at the time of recurrence			
III	8	27	0.35^a^
IV	16	34	
Histology			
Adenocarcinoma	14	29	0.06^a^
Squamous cell carcinoma	8	28	
Large cell carcinoma	2	1	
Others	0	3	
*EGFR* mutation status			
Mutant	3	8	0.51^a^
Wild-type	8	13	
Unknown	13	40	
Smoking history			
Current or former	20	45	0.44^a^
Never	3	9	
Unknown	1	7	
Number of treatment cycles, median (range)	2 (1–6)	2 (1–9)	0.50^b^
Response to prior chemoradiotherapy			
Complete response	1	0	<0.05^a^
Partial response	10	47	
Stable disease	10	14	
Progressive disease	3	0	
Time since prior chemoradiotherapy			
<6 months	10	19	0.36^a^
≥6 months	14	42	
Radiation dosage, median (range), Gy	60 (58–70)	60 (40–74)	0.52^b^
Prior chemotherapy regimen			
CDDP + VNR	5	20	0.17^a^
CDDP + S1	7	16	
CBDCA + PTX	6	17	
Others	6	8	
Number of regimens after progression following second-line chemotherapy			
0/1/2/≥3	11/10/3/0	12/22/12/15	
Median (range)	1 (0–2)	1 (0–6)	<0.05^b^

*Abbreviations:* EGFR, epidermal growth factor receptor; CDDP, cisplatin; VNR, vinorelbine; CBDCA, carboplatin; PTX, paclitaxel.

^*a*^Fisher’s exact test; ^*b*^ *Wilcoxon* rank-sum test.

**Table 2:** **Chemotherapy regimens used after progression following second-line chemotherapy**

	**Platinum combination therapy**		**Docetaxel monotherapy**	
	**Third-line**	**≥Fourth-line**	**Third-line**	**≥Fourth-line**
EGFR-TKI				
Gefitinib	1	1	11	5
Erlotinib	2	0	5	7
Single agent				
Docetaxel	0	1	-	1
Pemetrexed	2	0	2	3
Amrubicin	2	1	8	11
Gemcitabine	0	1	16	8
S1	0	0	5	12
Others	2	0	1	5
Platinum combination	3	0	0	5
Investigational agent	0	0	1	4

*Abbreviations:* EGFR-TKI, epidermal growth factor receptor tyrosine kinase inhibitor.

### Objective tumor response to therapy and survival

Objective tumor response is shown in Table 3. The differences in the response rate (RR) and disease-control rate between the two groups were not statistically significant (RR, *p* = 0.09; disease-control rate, *p* = 0.82).

**Table 3:** **Objective tumor response**

	**Platinum combination therapy (n = 24) n (%)**	**Docetaxel monotherapy (n = 61) n (%)**	***p*** value*
Complete response	0 (0)	1 (1.6)	0.30
Partial response	4 (16.7)	3 (4.9)	
Stable disease	10 (41.7)	31 (50.8)	
Progressive disease	9 (37.5)	24 (39.3)	
Not evaluable	1 (4.1)	2 (3.3)	
Response rate (%)	16.7	6.6	0.09
Disease control rate^a^ (%)	58.3	57.4	0.82

*Fisher’s exact test.

^a^Complete response + partial response + stable disease.

**Figure 1:**
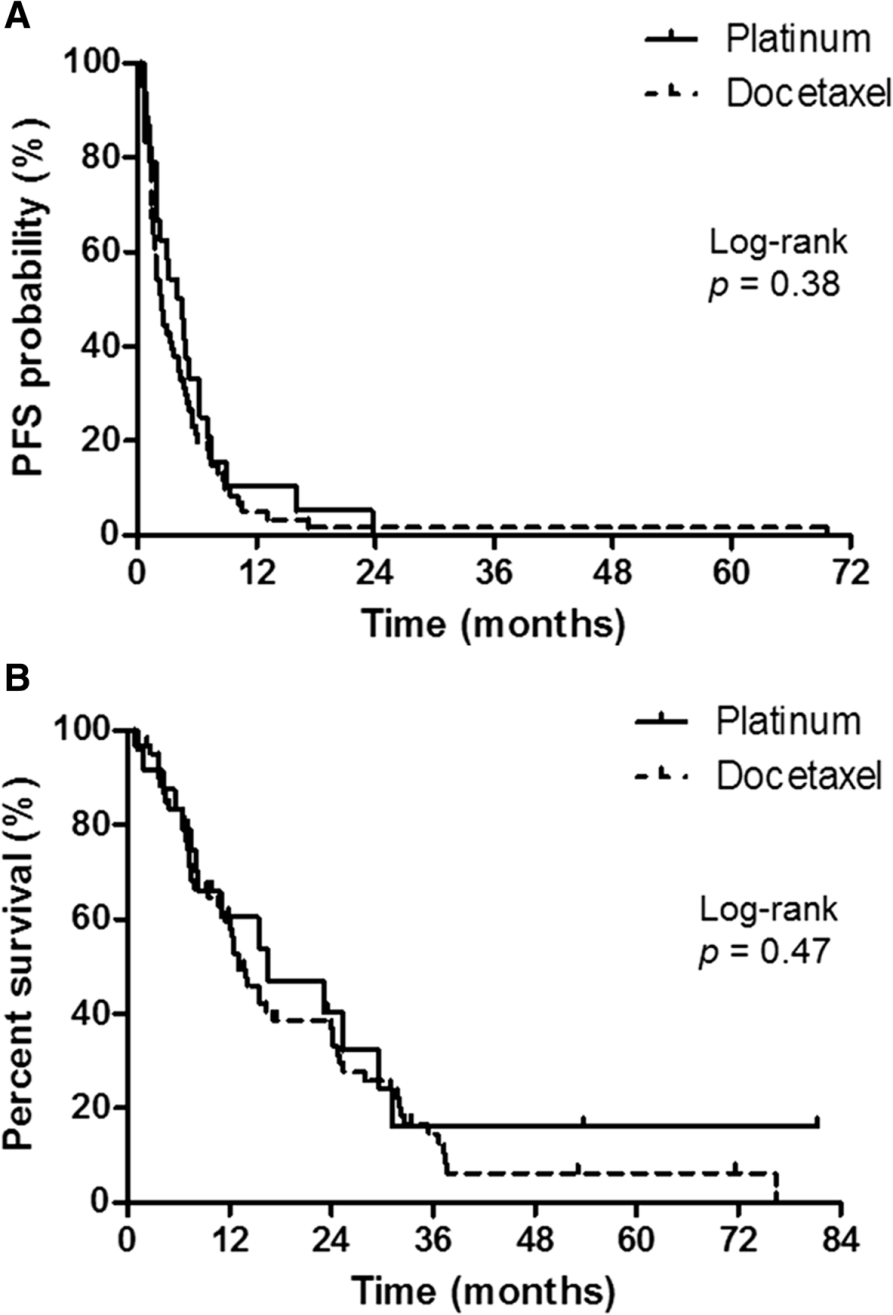
**Progression-free survival (PFS) and overall survival (OS) by treatment arm. (A)** PFS: platinum combination, median PFS = 4.2 months; docetaxel monotherapy, median PFS = 2.3 months. **(B)** OS: platinum combination, median OS = 16.5 months; docetaxel monotherapy, median OS = 13.0 months.

Progression-free survival (PFS) was similar between the platinum combination therapy group and the docetaxel monotherapy group (median, 4.2 vs. 2.3 months; hazard ratio [HR] = 0.81; 95% confidence interval [CI] = 0.51–1.29; *p* = 0.38) (Figure 1A). OS was also similar between the two groups (median, 16.5 vs. 13.0 months; HR = 0.82; 95% CI = 0.47–1.41; *p* = 0.47) (Figure 1B).

**Figure 2:**
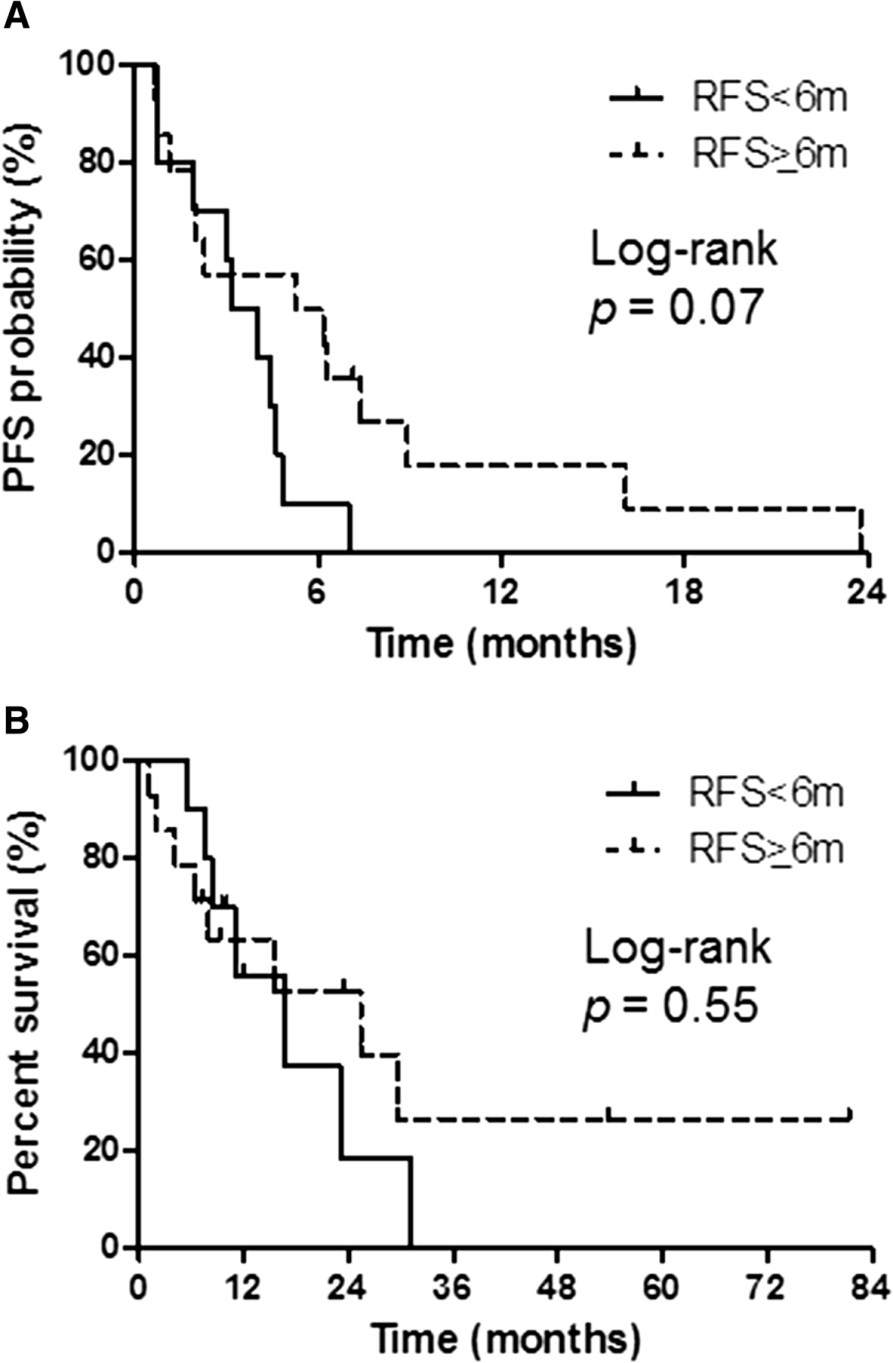
**Progression-free survival (PFS) and overall survival (OS) according to relapse-free survival (RFS) in the platinum combination therapy group. (A)** PFS: RFS <6 months, median PFS = 3.6 months; RFS ≥6 months, median PFS = 5.6 months. **(B)** OS: RFS <6 months, median OS = 16.5 months; RFS ≥6 months, median OS = 25.3 months.

**Figure 3:**
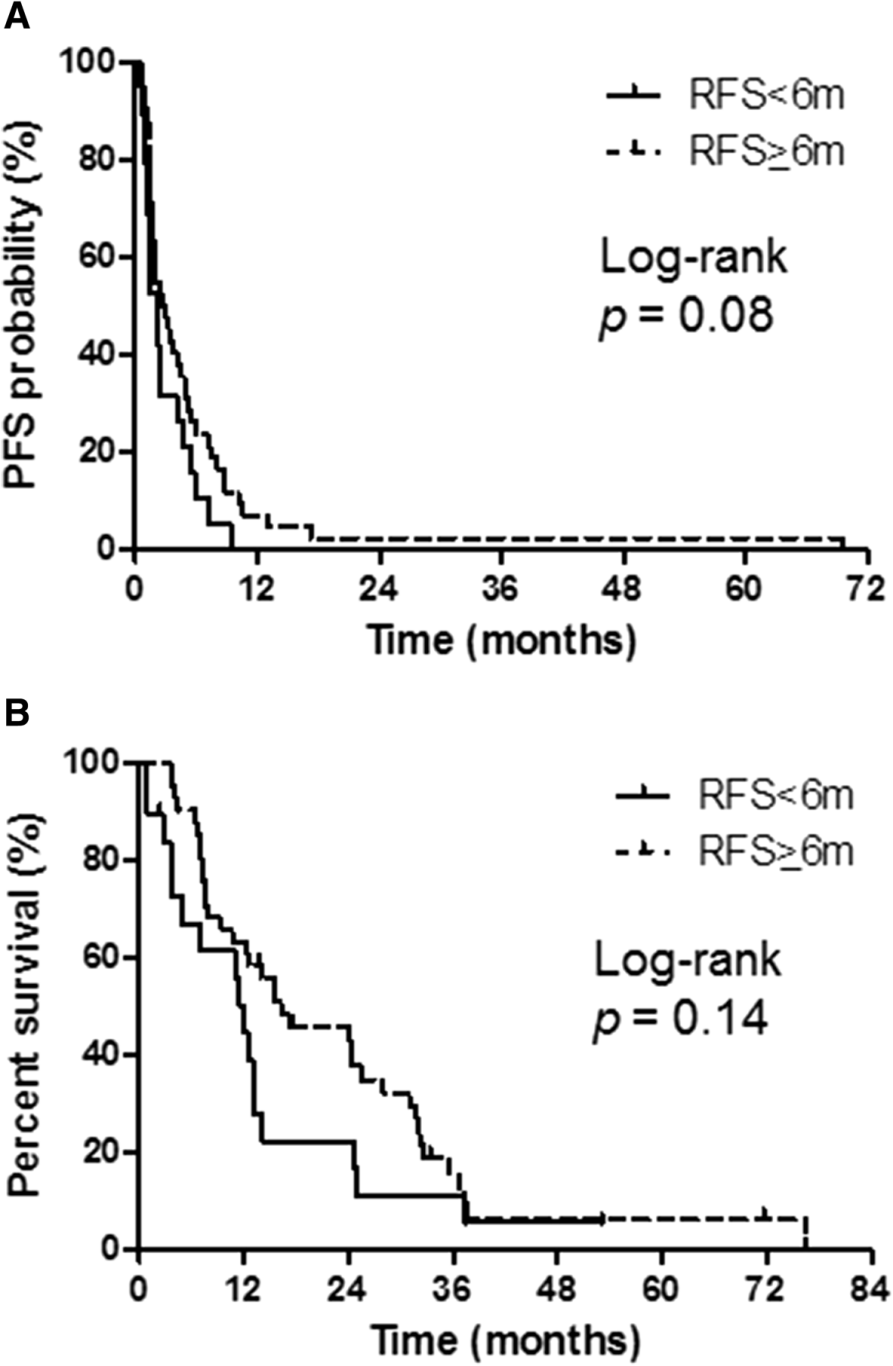
**Progression-free survival (PFS) and overall survival (OS) according to relapse-free survival (RFS) in the docetaxel monotherapy group. (A)** PFS: RFS <6 months, median PFS = 2.2 months; RFS ≥6 months, median PFS = 2.8 months. **(B)** OS: RFS <6 months, median OS = 12.0 months; RFS ≥6 months, median OS = 16.3 months.

Patients in each treatment group were further stratified based on relapse-free survival (RFS). In the platinum combination therapy group, 10 patients had an RFS of <6 months, and 14 had an RFS of ≥6 months. Among the platinum combination therapy patients with an RFS of <6 months, one had a partial response (PR); four had stable disease (SD), and three experienced progressive disease (PD). Among the platinum combination therapy patients with an RFS of ≥6 months, three had a PR; six had SD, and five had PD. The median PFS for the platinum combination therapy patients with an RFS of <6 months and ≥6 months was 3.6 and 5.6 months, respectively (log-rank, *p* = 0.07) (Figure 2A). Furthermore, the corresponding median OS for these patients was 16.5 and 25.3 months, respectively (log-rank, *p* = 0.55) (Figure 2B). In the docetaxel monotherapy group, 19 patients had an RFS of <6 months, and 42 had an RFS of ≥6 months. Among the docetaxel monotherapy patients with an RFS of <6 months, one had a PR; eight had SD, and seven had PD, whereas among those with an RFS of ≥6 months, two had a complete response/PR; 23 had SD, and 17 had PD. The median PFS for the docetaxel monotherapy patients with an RFS of <6 months and ≥6 months was 2.2 and 2.8 months, respectively (log-rank, *p* = 0.08) (Figure 3A). Furthermore, the corresponding median OS for these patients was 12.0 and 16.3 months, respectively (log-rank, *p* = 0.14) (Figure 3B). The differences in median PFS and OS among the four sub-groups (platinum combination therapy patients with an RFS of ≥6/<6 months and docetaxel monotherapy patients with an RFS of ≥6/<6 months) were not statistically significant.

### Toxicity

A summary of the toxicities observed in both groups is shown in Table 4. Hematological toxicities, particularly leukopenia and neutropenia, tended to be more frequent in the docetaxel monotherapy group. However, there were no statistically significant differences between the two groups in grade 3 and above leukopenia (*p* = 0.06) or grade 3 and above neutropenia (*p* = 0.08). The incidences of other toxicities did not differ between the two groups. Discontinuation of chemotherapy related to toxicity occurred in one patient in the platinum combination therapy group (grade 4 leukopenia) and five patients in the docetaxel monotherapy group (grade 3 interstitial pneumonitis in two patients, grade 3 febrile neutropenia in one patient, grade 3 paralytic ileus in one patient, and grade 3 edema in one patient). All grade ≥3 adverse events related to platinum-based chemotherapy, such as nausea, vomiting, nephrotoxicity, neurotoxicity, and fatigue, were not observed in more than 5% of the patients. There were no treatment-related deaths in either group.

**Table 4:** **CTCAE grade ≥3 adverse events observed in more than 5% of patients**

	**Platinum combination therapy (n = 24)**			**Docetaxel monotherapy (n = 61)**			
	**Grade 3**	**Grade 4**	**Total (%)**	**Grade 3**	**Grade 4**	**Total (%)**	***p*** value*
Leukocytopenia	8	2	10 (41.6)	30	9	39 (63.9)	0.06
Neutropenia	8	6	14 (58.3)	16	28	44 (72.1)	0.08
Anemia	3	0	3 (12.5)	2	0	2 (3.3)	0.12
Febrile neutropenia	2	0	2 (8.3)	6	0	6 (9.8)	0.82
Anorexia	1	0	1 (4.2)	5	0	5 (8.2)	0.49
Infection	1	0	1 (4.2)	3	0	3 (4.9)	0.88

*Abbreviations:* CTCAE, Common Terminology Criteria for Adverse Events version 3.0.

*Fisher’s exact test.

## Discussion

Platinum-based doublet chemotherapy prolongs survival and improves quality of life in patients with a PS of 0–2. Chemotherapy should be initiated while the patient maintains a good PS (Reck et al. 2014). Such a recommendation is based on findings from previous trials on metastatic diseases, including both primary metastatic and relapsed diseases after local therapies.

Platinum resistance is often a concern when second-line treatment for relapsed NSCLC is initiated after failure of first-line treatment (Huisman et al. 2000). As previously discussed, prior exposure to platinum as part of CRT might reduce the likelihood of response to subsequent platinum therapy. In fact, known molecular mechanisms of platinum resistance may be responsible for the worse outcomes observed in patients previously treated with platinum-based CRT (Cosaert and Quoix 2002). The 10% RR observed by Paramanathan *et al.* in patients who were re-challenged with platinum-based chemotherapy at disease relapse, supports the platinum-resistance hypothesis (Paramanathan et al. 2013).

However, a small retrospective study of patients treated with first-line chemotherapy and re-challenged on relapse with the same chemotherapy (usually platinum-based) showed a response rate of 29% and superior survival compared to those treated with a second-line agent (docetaxel). Such a result suggests that in certain cases, re-challenging with the first-line agents, rather than switching to a different drug, is a reasonable strategy (Nagano et al. 2010). Furthermore, if this study limits its enrolled patients to only responders as the previous study did, it might have the same outcome. However, no phase III trials have been conducted in a second-line setting to evaluate the value of platinum doublets, including newer agents such as taxanes and pemetrexed, for the treatment of NSCLC. Recently, two meta-analyses evaluated pemetrexed- and docetaxel-based doublets as second-line treatments and showed an advantage in RR and PFS compared to treatment with single agents (Qi et al. 2012a; Qi et al. 2012b). In these meta-analyses, only two trials included platinum agents, and only one of these studies enrolled patients who had previously been treated with platinum agents. This recent study by Ardizzoni *et al.* was a pooled analysis of two randomized phase II trials comparing carboplatin/pemetrexed and pemetrexed alone to treat stage IV NSCLC pre-treated with platinum agents (Ardizzoni et al. 2012). Overall, the RR significantly increased from 9% to 15%, and PFS from 3 to 3.9 months in the combination arm, but the OS was similar in second-line treatment. Our study showed a 16.7% tumor RR for the platinum combination therapy group, which was comparable to the RR reported by the above-mentioned study.

Our results indicated that the treatment protocol for relapsed NSCLC in patients previously treated with platinum-based CRT might require a re-evaluation. A recent meta-analysis showed that similar one-year survival rates were achieved with non-platinum-based doublets as first-line therapy (D’Addario et al. 2005). Published treatment guidelines from the American Society of Clinical Oncology suggest that non-platinum-based doublets might provide an alternative option to certain groups of patients (Azzoli et al. 2011). Given the higher toxicities experienced by patients receiving platinum-based doublet therapy and the similar survival rates for both platinum and non-platinum-based doublet therapy, it would be reasonable to advocate a change in treatment protocol for this particular group of patients. Alternatively, these patients could be treated with a non-platinum single agent such as pemetrexed, docetaxel, or erlotinib, depending on the clinical situation.

Many previous prospective trials and retrospective studies have not sufficiently documented patients’ prior treatment history, including platinum exposure. Moreover, although patients who relapse after prior CRT are commonly seen in clinical practice, recent trials of first-line chemotherapy for metastatic disease have excluded them. Given the potential for treatment resistance, a history of prior platinum exposure should be collected from patients enrolled in future trials of chemotherapy for metastatic disease. Although we previously reported that re-challenge with platinum combination chemotherapy was effective and safe for relapsed patients after postoperative cisplatin-based adjuvant chemotherapy for resected NSCLC (Imai et al. 2013), our current report suggests that non-platinum based agents might be considered in NSCLC patients who relapse after platinum-based CRT. Although both studies consider prior treatment history, the optimal treatment following CRT may differ from the one following postoperative adjuvant chemotherapy.

In the present study, although 23 of the 85 patients did not receive subsequent chemotherapy after progressing past second-line chemotherapy, many others did. The increased number of treatment regimens currently used following progression after second-line chemotherapy might be the result of more active compounds, such as gefitinib, erlotinib, docetaxel, pemetrexed, amrubicin, gemcitabine, and S-1, available for the treatment of advanced NSCLC. In fact, a number of different compounds were used to treat our patients, as shown in Table 2. It might be of importance to carefully examine the correlation between epidermal growth factor receptor tyrosine kinase inhibitor (EGFR-TKI) responders or additional therapies and survival. However, statistical analysis might be challenging to perform because the number of patients receiving EGFR-TKI is relatively small. Furthermore, response to EGFR-TKI reportedly depends on patients’ ethnicity, sex, smoking history, and histological type according to a previous study on patients receiving gefitinib based on clinical background (Mitsudomi et al. 2006). In addition, although analysis of *EGFR* mutation status is routinely performed for NSCLC patients currently, it was not previously, which might affect patient stratification for EGFR-TKI treatment.

The interval from first- to second-line chemotherapy was quite variable in both of our study groups. Considering only patients whose RFS was more than six months, the median PFS was 5.6 months and 2.8 months (*p* = 0.30), and the median OS was 25.3 months and 16.3 months (*p* = 0.44) in the platinum combination and docetaxel groups, respectively. Although these differences were not significant, there was a trend toward longer PFS and OS in this group. It is known that the treatment efficacy for metastatic cervical cancer is similarly low to that seen in our study if platinum-based CRT has been given less than six months before treatment (Monk et al. 2009). Due to the small number of patients in our study with an RFS of less than six months, these results might not be significantly different. The fact that the median PFS was worse for patients relapsing within six months of prior platinum-based CRT could relate to the more aggressive nature of relapsed disease or to a potential resistance to chemotherapy. Given the retrospective nature of this study, we could not determine the causative mechanism behind these results, and future prospective research is needed.

Patients who were re-challenged with platinum combination and those receiving docetaxel monotherapy had similar grades and incidences of toxicity, suggesting that the cumulative toxicity of platinum might be absent. In particular, toxicities such as hearing impairment, neurotoxicity, renal dysfunction, and allergic reactions caused by repeated use of platinum combination chemotherapy re-challenge were not notable. In the docetaxel group, however, hematological toxicities, especially leukopenia and neutropenia, were more frequent. A possible reason for this finding might be that the reported grade of adverse event was the worst value observed, and patients receiving docetaxel continued their treatment until PD.

This study has several limitations. First, it was a retrospective analysis, and the toxicities might have been underestimated. Reducing, skipping, or delaying the planned chemotherapy was allowed at the discretion of the attending physician. To minimize this potential bias, all consecutive patients treated at our institutes were included in the analysis, and the patients’ original charts were thoroughly reviewed. Second, the sample size of our study was small. Nonetheless, we believe that the results of the present investigation are worthwhile because this small sample size is indicative of the relatively small population of patients who relapse after platinum-based CRT. Finally, the sample size of the two groups in this study was markedly different, but the patients’ demographic data were well-balanced.

## Conclusion

We did not find platinum combination re-challenge therapy to be superior in efficacy for relapsed patients previously treated with platinum-based CRT, suggesting that if an NSCLC patient relapses after receiving platinum-based CRT, it is not necessary to favor the use of platinum combination chemotherapy. To our knowledge, this is the first study to evaluate and compare the efficacy and safety of platinum combination re-challenge therapy and docetaxel monotherapy in NSCLC patients previously treated with platinum-based CRT. Although the results of our investigation might contribute to a better understanding of the clinical benefit of platinum combination chemotherapy re-challenge after NSCLC recurrence, future trials comparing platinum and non-platinum chemotherapy following relapse after CRT are needed to help guide clinical practice and recommendations.
